# Germanium-Vacancy Single Color Centers in Diamond

**DOI:** 10.1038/srep12882

**Published:** 2015-08-07

**Authors:** Takayuki Iwasaki, Fumitaka Ishibashi, Yoshiyuki Miyamoto, Yuki Doi, Satoshi Kobayashi, Takehide Miyazaki, Kosuke Tahara, Kay D. Jahnke, Lachlan J. Rogers, Boris Naydenov, Fedor Jelezko, Satoshi Yamasaki, Shinji Nagamachi, Toshiro Inubushi, Norikazu Mizuochi, Mutsuko Hatano

**Affiliations:** 1 Department of Physical Electronics, Tokyo Institute of Technology, Meguro, Tokyo 152-8552, Japan; 2 CREST, Japan Science and Technology Agency, Chiyoda, Tokyo; 3 Graduate School of Engineering Science, Osaka University, Toyonaka, Osaka 560-8531, Japan; 4 Nanomaterials Research Institute, National Institute of Advanced Industrial Science and Technology, Tsukuba, Ibaraki 305-8568, Japan; 5 Institute for Quantum Optics and Center for Integrated Quantum Science and Technology (IQst), Ulm University, Albert-Einstein-Allee 11, Ulm D-89081, Germany; 6 Advanced Power Electronics Research Center, National Institute of Advanced Industrial Science and Technology, Tsukuba, Ibaraki 305-8568, Japan; 7 Nagamachi Science Laboratory, Amagasaki, Hyogo 661-0976, Japan; 8 Shiga University of Medical Science, Otsu, Shiga 520-2192, Japan

## Abstract

Atomic-sized fluorescent defects in diamond are widely recognized as a promising solid state platform for quantum cryptography and quantum information processing. For these applications, single photon sources with a high intensity and reproducible fabrication methods are required. In this study, we report a novel color center in diamond, composed of a germanium (Ge) and a vacancy (V) and named the GeV center, which has a sharp and strong photoluminescence band with a zero-phonon line at 602 nm at room temperature. We demonstrate this new color center works as a single photon source. Both ion implantation and chemical vapor deposition techniques enabled fabrication of GeV centers in diamond. A first-principles calculation revealed the atomic crystal structure and energy levels of the GeV center.

Color centers in diamond are promising candidates for single photon sources which are a valuable resource for quantum cryptography^1^ and quantum information processing^2^. Although many optically active structures have been found in diamond^3^, only a limited number have been reported as a single photon source^4^,^5^, such as Nitrogen-vacancy (NV)^6^,^7^,^8^, Silicon-vacancy (SiV)^9^,^10^, NE8^11^, and Cr-related^12^ centers. Of these, only the NV and SiV centers have been reproducibly fabricated^13^. To achieve superior optical properties and to more deeply understand the formation mechanism and characteristics of color centers in diamond, further exploration of novel color centers which can be reproducibly formed and can emit single photons is required. In this study, we demonstrate that a germanium-related complex fabricated in diamond shows a sharp and strong luminescence band with a zero phonon line (ZPL) at around 602 nm, and has single photon emission capability at room temperature. Using first principle calculations, we found this color center to be composed of a Ge atom and a vacancy, namely GeV center, with the Ge atom relaxing to the bond-centered position giving D_3d_ symmetry as in the SiV center. As well as production by ion implantation, we also demonstrate the formation of the GeV centers in diamond by chemical vapor deposition (CVD) and show that this leads to narrower line widths and smaller variation of the peak position. Theoretical calculation of the expected energy levels has revealed the reason for fluorescence energy difference from the SiV center.

## Results

**Figure 1:**
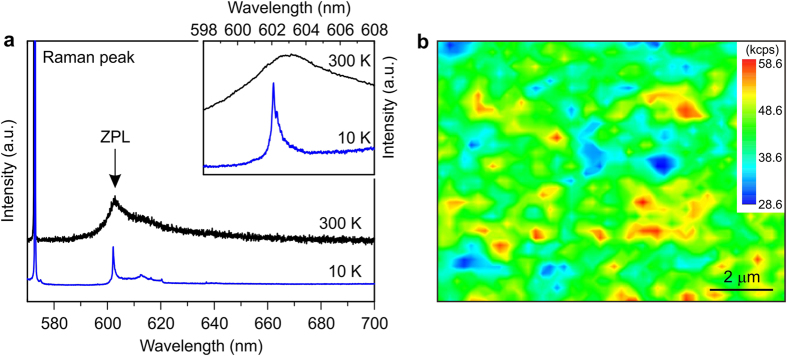
Luminescence characteristics of GeV color center in diamond formed by ion implantation. (**a**) PL spectra from a Ge ion implanted diamond at 300 K and 10 K. The inset shows ZPL at both the temperatures. (**b**) Intensity mapping of the ZPL between 595 and 608 nm at room temperature. The Ge ions were implanted to give a peak concentration of 1 × 10^19^ cm^−3^. The ion implantation conditions were determined by simulating SRIM^14^. The measurements were done by using a micro-Raman system at 300 K and a micro-PL system at 10 K.

Figure 1a shows a PL spectrum from a Ge ion-implanted diamond at room temperature. A peak was found at 602.7 nm (~2.06 eV), accompanying a Raman scattering peak from the diamond substrate. We confirmed that the peak appeared over the whole substrate surface although the intensity varied (Fig. 1b) and that the peak intensity increased with increasing the Ge ion implantation dose (see Supplementary Information Fig. S1). It should be noted that this peak was not observed by ion implantation of different elements (see Supplementary Information Fig. S2). Thus, the peak at 602 nm was concluded to be the ZPL of the Ge-related structure in diamond. The full width at half maximum (FWHM) of the peak decreased at 10 K, where the ZPL splits into two components with an energy separation of 0.67 meV (inset in Fig. 1a). Other lines seem to appear around the two peaks, but a more detailed study would be necessary to determine the fine structure. This luminescence band was only visible after the high-temperature treatment at 800 °C and above. Ge ion implantation alone did not lead to the appearance of the peaks (see Supplementary Information Fig. S3). This fact indicates that the Ge forms a complex in diamond with a vacancy or vacancies diffusing during the high temperature annealing process, like other color centers related to vacancies^4^.

**Figure 2:**
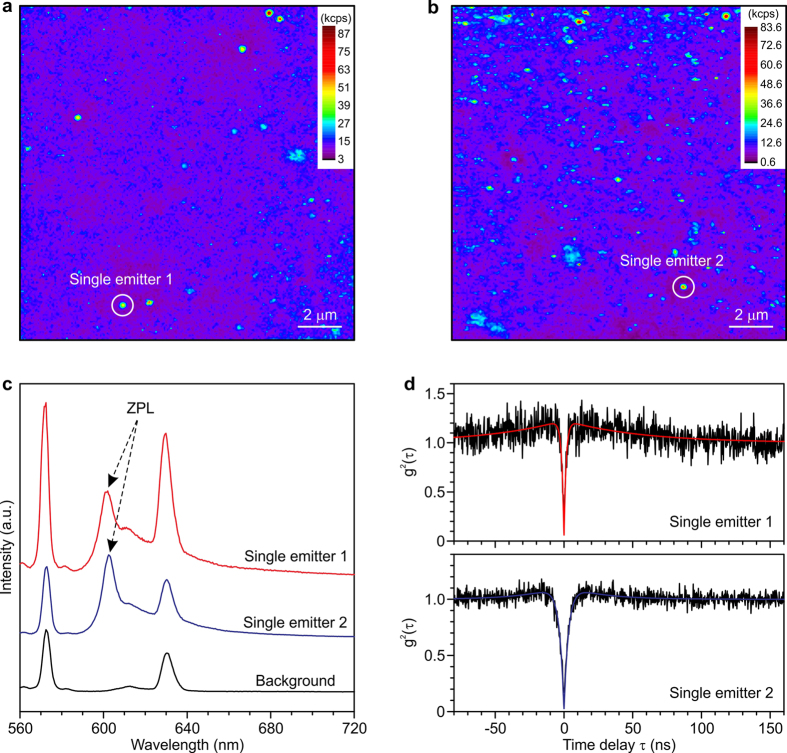
GeV single photon source. (**a,b**) Intensity mappings of GeV single emitters. (**c**) PL spectra and (**d**) g^2^(τ) function of the two GeV single centers, marked in the white circles in panels a and b. The background PL was collected at a position without a GeV center. The PL spectra were measured at an excitation laser power of 3 mW. The spectra from the single emitters were normalized at the ZPL. The g^2^(τ) functions were measured at an excitation laser power of 1 mW. The solid lines in panel d denote the fitting. The sample was prepared by ion implantation at a dose of 3.5 × 10^8^ cm^−2^ and an ion energy of 150 keV, leading to a projected range of 57 nm from the surface. All the measurements were performed by using a confocal microscope system at room temperature. The confocal images and g^2^(τ) functions were observed with a band-pass filter of 25 nm FWHM around 600 nm.

Here, we demonstrate that the GeV color center works as a single photon emitter. Figure 2a,b show PL maps of a sample prepared by ion implantation with a much lower peak concentration (1 × 10^14^ cm^−3^). A number of nanometer-sized fluorescent spots are observed in both the images. Two individual spots with a size of around 350 nm, marked in the white circles, show clear ZPLs from the GeV center, having peak positions of 601.6 and 602.4 nm for emitter 1 and 2, respectively (Fig. 2c). We measured the second-order autocorrelation function g^2^(τ)^4^,^8^ for these spots with a band-pass filter around the ZPL from the GeV center (Fig. 2d). Sharp dips at a delay time τ of 0 ns for both the emitters indicate antibunching, and g^2^(0) below 0.5 is proof of single photon emission^15^ from the GeV centers. The g^2^(τ) data were fitted with the equation for a three-level system^13^, 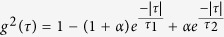, where τ_1_, τ_2_, and α are the fitting parameters. We obtained a value of 1.4–5.5 ns for τ_1_, which provides an estimate of the excited state lifetime. This is much shorter than the NV centers (~12 ns)^6^,^16^ and comparable to the SiV centers (1.1–2.4 ns)^9^ in the bulk diamond.

**Figure 3:**
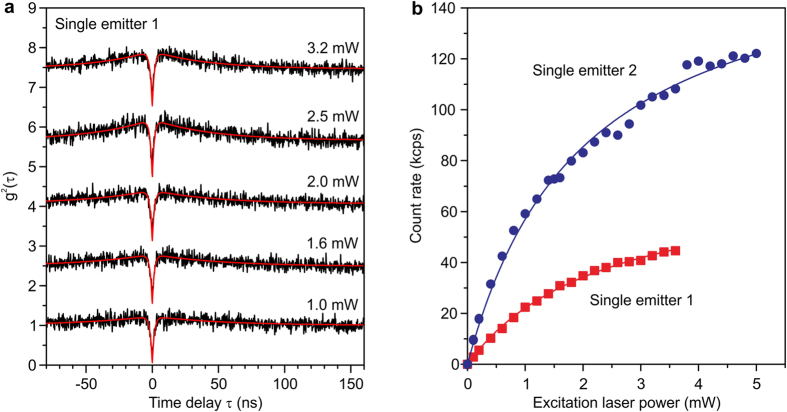
Analysis of GeV single photon source. (**a**) The g^2^(τ) functions measured with different laser powers from 1.0 to 3.2 mW. The lines were shifted vertically for clarity. (**b**) Saturation characteristics of the two GeV single centers. The background was subtracted. The solid lines in panels a and b denote the fitting. All the measurements were performed by a confocal microscope system at room temperature with a band-pass filter of 25 nm FWHM around 600 nm.

To analyze the behavior of the GeV single photon source in more detail, we measured the excitation power dependence of the g^2^(τ) function (Fig. 3a). The antibunching was observed at the various laser powers, while increased excitation power led to the generation of maximum points over the unity. This bunching suggests that storage mechanisms are present in the GeV center, such as a shelving state or photo-ionization. The photon count rate is an important figure of merit for single photon sources. Saturation curves of these two emitters are shown in Fig. 3b. Fits of the form, *I* *=* *I*_*∞*_ × *P/*(*P* + *P*_*sat*_)^9^, where *P*_*sat*_ is the saturation power, reveal saturation intensities *I*_*∞*_ of 75 kcps (emitter 1) and 170 kcps (emitter 2). The value from the emitter 2 is slightly lower than 200 kcps reported for a SiV single emitter fabricated by CVD^17^. However, we measured the fluorescence only in the band of about 25 nm width around 600 nm due to a band-pass filter being required to avoid Raman signals around ZPL. It is expected that *I*_*∞*_ for GeV would be increased if the fluorescence could be detected across a wider spectral region. Therefore, the luminescence intensity of GeV is considered to be comparable to that of SiV. It should be noted that *I*_*∞*_ of NV^−^ in the setup described in Ref. 17 is similar to that in the present measurement setup, making the comparison here between the GeV and SiV centers independent of the measurement system.

**Figure 4:**
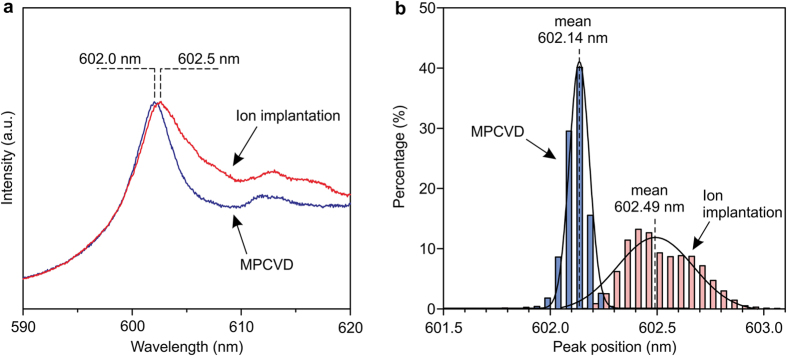
MPCVD-incorporated GeV color center ensemble. (**a**) PL spectrum from the MPCVD-incorporated GeV centers, compared with ones fabricated by ion implantation. (**b**) Histograms of the ZPL position of the GeV centers fabricated by MPCVD and ion implantation.

CVD incorporation of color centers is an important technique to obtain high-quality fluorescence centers in diamond without implantation damages^10^,^17^,^18^. Here, we demonstrate the formation of GeV color centers in diamond by microwave plasma CVD (MPCVD). A diamond film was grown on a single-crystal diamond substrate by MPCVD with a Ge solid source. The Ge incorporation in the diamond film was confirmed by secondary ion mass spectrometry (SIMS) (see Supplementary Information Fig. S4). Figure 4a shows a PL spectrum from the CVD-incorporated GeV centers, possessing a narrower line width of 4–5 nm than that (6–7 nm) of the GeV centers formed by ion implantation and annealing. Histograms of the ZPL position for GeV centers in the MPCVD and ion implantation samples are shown in Fig. 4b, and there is a slight blue-shift in the MPCVD sample. The CVD-prepared GeV centers also have a narrower inhomogeneous distribution (σ = 0.05 nm) than those produced by ion implantation (σ = 0.18 nm). These effects could arise from the lower defect density and lower strain in the sample prepared by MPCVD.

**Figure 5:**
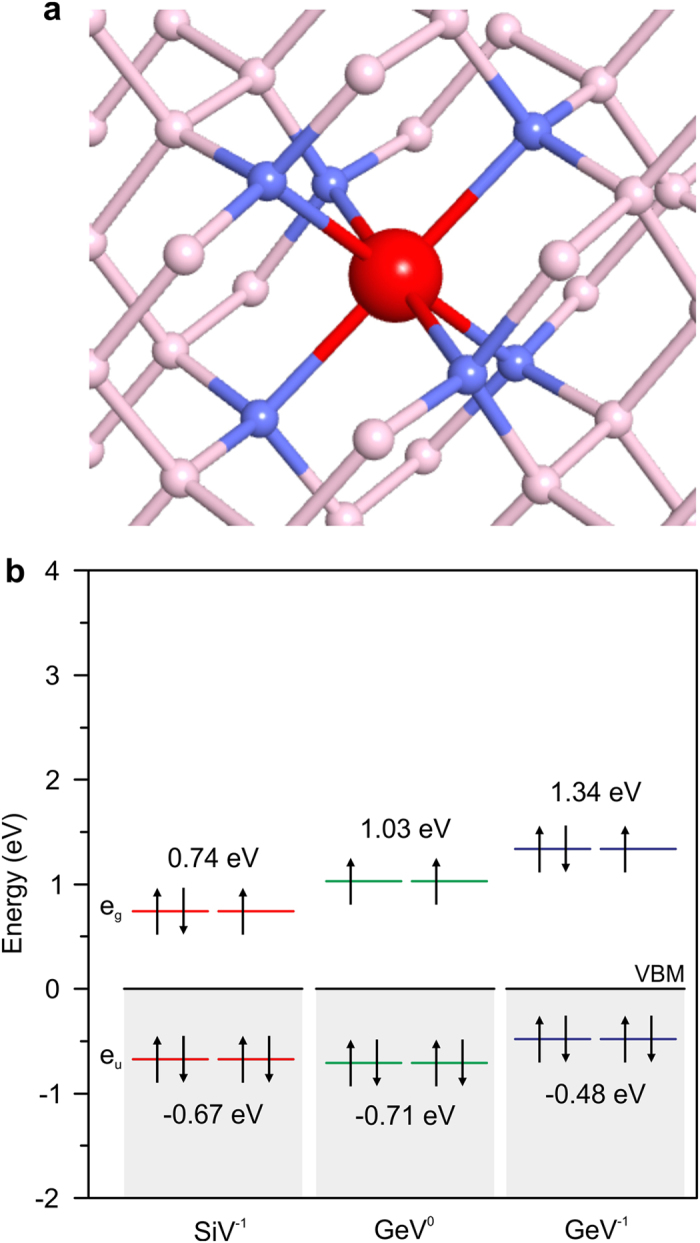
First principles calculation of GeV color center in diamond. (**a**) Crystal structure of the GeV color center in diamond. The red sphere denotes a Ge atom. The small blue and pink spheres are carbon atoms. The atomic structure shown is for the negatively charged state. (**b**) Energy levels of the SiV and GeV color centers in diamond. The energy was calculated with respect to VBM of diamond.

The crystal structure of the Ge-related color center was calculated from first-principles. First, we started from a structure assimilating to the NV center, i.e. one Ge atom at a substitutional site and one neighboring vacancy also at a carbon site. By performing structural relaxation, no potential barrier was found for the Ge atom on a trajectory from the substitutional site to an interstitial site between the lattice vacancies as shown in Fig. 5a, in agreement with previous theoretical study^19^. This geometry belongs to the symmetry group of D_3d_, and it is the same configuration as the SiV center^20^. These atoms have similar electron configurations, and both prefer the interstitial position as they are substantially larger than the carbon atoms of the diamond lattice.

Energy levels of the SiV and GeV color centers were calculated within the density functional theory (DFT) using PBE functional to investigate the origin of the fluorescence wavelength difference (SiV has a ZPL at 738 nm^21^). Optical matrix elements were calculated to check optical selection rule among the computed levels. The calculation was done for the negatively charged SiV (SiV^−1^)^22^,^23^, neutral GeV (GeV^0^), and negatively charged GeV (GeV^−1^) centers. Figure 5b shows calculated energy levels of the SiV and GeV color centers in diamond. For both centers, the e_u_ and e_g_ levels are doubly-degenerate and the e_g_ levels are partially occupied. The e_u_ levels positioned in the valence band of diamond are at −0.67 eV for SiV^−1^, −0.71 eV for GeV^0^, and −0.48 eV for GeV^−1^. Here, the energy is defined from the valence band maximum (VBM) of diamond. The e_g_ levels are, however, in the band gap of diamond. The e_g_ level of the SiV^−1^ center is +0.74 eV above VBM, which agrees well with the previous calculation^24^, while the GeV^0^ and GeV^−1^ centers have higher e_g_ energies. Here, no significant difference in the energy between the e_u_ and e_g_ levels were seen for the GeV^0^ and GeV^−1^ centers. Although the charge state of the GeV center is not clear in this study, the higher e_g_ levels should be the origin giving the higher fluorescence energy in the GeV center. Although the spin configuration is considered in the diagram according to Ref. 24, the current calculation was done under spin-unpolarized approximation. [Despite ignorance of the spin polarization in the present calculation and difference in the cell size (216 atoms in the present work and 512 atoms in Ref. 24) as well as k-point sampling (four k-points at the wedge of the whole Brillouin zone in the present work and Γ point in Ref. 24), the agreement in the e_u_ and e_g_ energy levels between the present calculation and Ref. 24 are satisfactory good. We thus judge that quantitative results provided by the present computational scheme is enough to discuss impurity levels].

## Discussion

We have demonstrated that GeV centers can be reliably and reproducibly fabricated in diamond by ion implantation under various implantation conditions. Importantly, the capability of the single photon emission has been demonstrated. Additionally, it was confirmed that GeV centers can form by the incorporation of germanium during MPCVD growth, and these show less variation of the ZPL peak positions. These results establish the GeV center as a new single photon emitter that can easily be formed in diamond. Here, the GeV centers were fabricated in the bulk and thin film diamonds, but the morphology and size of diamond is in principle not limited. For example, the incorporation of the GeV centers in nanodiamonds should be possible, which is important for bio-labelling applications^25^,^27^.

The large inhomogeneous distribution of the fluorescence wavelength of the GeV centers produced by ion implantation (shown in Fig. 4b) likely originates from the strain of the GeV complex structure in the diamond lattice. It is hard to completely remove residual defects, such as interstitial C atoms and vacancies, created during ion implantation by annealing. The remaining defects cause displacement and distortion of the atomic crystal structure of the GeV center, which would alter the energy levels and thus the peak position^27^. We evaluated eleven GeV single photon emitters in this study. Four of them possessed peak positions largely shifted from 602 nm (see Supplementary Information Fig. S5), which should be caused by the implantation damages. The difference in the count rate in Fig. 3b occurs probably for the same reason. Two approaches can be considered to overcome the problem. (1) In the same manner as the ensemble, the MPCVD fabrication would provide GeV single photon sources with a uniform peak position and a narrower line width, potentially achieving indistinguishability as demonstrated for the SiV centers^28^. (2) A higher temperature annealing after ion implantation can reduce the damages. Our preliminary studies show it has a positive effect on the GeV center ensemble, but also suggest that further optimization is required (see Supplementary Information Section A).

From the viewpoint of the crystal structure and measured luminescent characteristics, the GeV and SiV centers have the similar features as a photon source. It is, however, expected that the GeV center has an advantage of more controlled fabrication by MPCVD. Even though the Ge solid source was placed with the diamond substrate on the sample holder and a Si source was not introduced intentionally during the MPCVD growth in this study, the concentration of Si in the diamond film was higher than that of Ge (see Supplementary Information Fig. S4). Silicon is a common contaminant due to its presence in silica parts such as windows. It is also possible that the larger atomic size of the Ge atom might reduce the incorporation efficiency into diamond compared with the Si atoms. This fact suggests that the GeV centers have a potential to be fabricated with high controllability for the scalable single photon sources, and could be less affected by the unintentional doping.

In summary, we have discovered a novel GeV color center in diamond and demonstrated it as a single photon emitter at room temperature with a ZPL at around 602 nm and an estimate of an excited-state lifetime of about 1.4 ns. The ion implantation technique and subsequent high-temperature anneal formed both ensemble and single photon emitters of the GeV centers, while diamond growth by MPCVD with a Ge crystal enabled us to fabricate high-quality GeV center ensemble. The first-principles calculation predicted that the GeV center has the same split-vacancy crystal structure as the SiV center, but shows the emission with the shorter wavelength resulting from the higher e_g_ state in the energy level in the GeV center.

## Methods

### Sample preparation

The luminescent Ge-related structure was prepared by ion implantation of Ge ions into diamond and subsequent annealing or CVD. High purity IIa-type (001) single-crystal diamond substrates (Element six, electronic grade) were used for the ion implantation experiments. The amount of nitrogen impurities in the substrate is below 5 ppb. Implantation of the Ge ions was performed by an ion implantation system over whole the surface at room temperature. The ion implantation energy ranged from 150 to 260 keV, and the Ge ion doses were 3.5 × 10^8^ – 5.9 × 10^13^ cm^−2^. The Ge ions (^70^Ge or ^73^Ge) were implanted after mass separation. Subsequently, the samples were annealed at 800 °C for 30 min. The CVD growth was performed by a MPCVD system with a spherical-form resonator. A gas mixture of H_2_ (198 sccm) and CH_4_ (2 sccm) was used for the diamond film growth, and a Ge crystal was placed together with a (111) diamond substrate (Sumitomo, Ib-type) on a Mo sample holder. The gas pressure and growth temperature were 6 kPa and 820 °C, respectively. The growth was performed for 2 h, leading to a diamond film thickness of about 100 nm.

### Optical measurement

The PL spectra and intensity mapping at room temperature were recorded by a micro-Raman system and a home-built confocal microscope set-up with an excitation wavelength of 532 nm. For the low temperature measurements at 10 K, a micro-PL system with an excitation wavelength of 532 nm was used. The g^2^(τ) function was measured using a Hanbury Brown-Twiss interferometer^28^ with two avalanche photo diode detectors. For the measurements of the confocal images and antibunching of the single centers, an oil-immersion objective (NA = 1.4) was used and a band pass filter (Edmund; 25 nm bandpass 600 nm) was placed in the detection channel to avoid the Raman signals around the ZPL.

### Theoretical calculation

For performing the first-principles calculations, we employed DFT^29^,^30^ with plane wave basis set. The exchange-correlation energy was expressed by the generalized gradient approximation (GGA) with PBE functional^31^. A 216-atom cell was used for the super-cell calculation. Four irreducible k-points including high-symmetric Γ, X, and L-points were used for momentum-space integration. Interaction of valence wavefunction and ions was expressed by Troullier-Martins type pseudopotentials^32^.

## Additional Information

**How to cite this article**: Iwasaki, T. *et al.* Germanium-Vacancy Single Color Centers in Diamond. *Sci. Rep.* **5**, 12882; doi: 10.1038/srep12882 (2015).

## Supplementary Material

Supplementary Information
