# Supplementary material for: Germanium-Vacancy Single Color Centers in Diamond
Source: Sci Rep. 2015 Aug 7;5:12882. doi: 10.1038/srep12882 (PMC4528202; doi:10.1038/srep12882)
Supplement: Supplementary Information [file srep12882-s1.doc]

**Supplementary Information**

**Germanium-Vacancy Single Color Centers in Diamond**

Takayuki Iwasaki,^1,2,*^ Fumitaka Ishibashi,^3^ Yoshiyuki Miyamoto,^2,4^ Yuki Doi,^3^ Satoshi Kobayashi,^3^ Takehide Miyazaki,^2,4^ Kosuke Tahara,^1^ Kay D. Jahnke,^5^ Lachlan J. Rogers,^5^ Boris Naydenov,^5^ Fedor Jelezko,^5^ Satoshi Yamasaki,^2,6^ Shinji Nagamachi,^7,8^ Toshiro Inubushi,^8^ Norikazu Mizuochi^2,3^ and Mutsuko Hatano^1,2^

^1^Department of Physical Electronics, Tokyo Institute of Technology, Meguro, Tokyo 152-8552, Japan

^2^CREST, Japan Science and Technology Agency, Chiyoda, Tokyo

^3^Graduate School of Engineering Science, Osaka University, Toyonaka, Osaka 560-8531, Japan

^4^Nanosystem Research Institute, National Institute of Advanced Industrial Science and Technology, Tsukuba, Ibaraki 305-8568, Japan

^5^Institute for Quantum Optics and Center for Integrated Quantum Science and Technology (IQst), Ulm University, Albert-Einstein-Allee 11, Ulm D-89081, Germany

^6^Energy Technology Research Institute, National Institute of Advanced Industrial Science and Technology, Tsukuba, Ibaraki 305-8568, Japan

^7^Nagamachi Science Laboratory, Amagasaki, Hyogo 661-0976, Japan

^8^Shiga University of Medical Science, Otsu, Shiga 520-2192, Japan

1. **Effect of the Ge dose on the PL intensity.**

The dependence of the emission intensity from the GeV centers on the Ge dose during ion implantation was investigated. Fig. S1 shows PL spectra with the Ge doses ranging from 2×10^12^ – 2×10^15^ cm^-2^, prepared in a polycrystalline diamond substrate (Element six) by a focused-ion beam system with an ion energy of 40 keV. Apparently, the higher Ge doses lead to the higher ZPL intensities at 602 nm. Annealing at 1000 ºC after ion implantation reduced the line width and histogram of the peak position compared to annealing at 800 ºC (shown in Fig. 1 and 4 in the main text). However, the surface of the diamond substrates was sometimes graphitized in part at this higher temperature, resulting in reduced and non-uniform fluorescence intensities. Thus, we note that the optimization of the annealing conditions is necessary in further study.


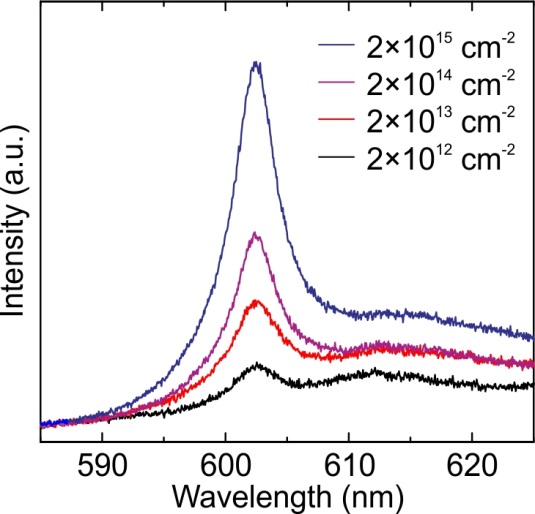


**Figure S1.** Dependence of the PL spectra on the Ge dose.

1. **Ion implantation of various elements into diamond.**

We have confirmed that the peak at 602 nm did not appear with ion implantation with different elements (Si, Cu, and Au) as shown in Fig. S2. Note that the peaks between 601 and 615 nm are second-order Raman signals from diamond^1^.The Si ion implantation led to a well-known ZPL at 738 nm, but no peak at around 602 nm. A large amount of defects were thought to be produced by the Cu and Au ion implantation. These treatments also did not result in the formation of the peak at 602 nm, which indicates that the peak is unambiguously related with Ge.


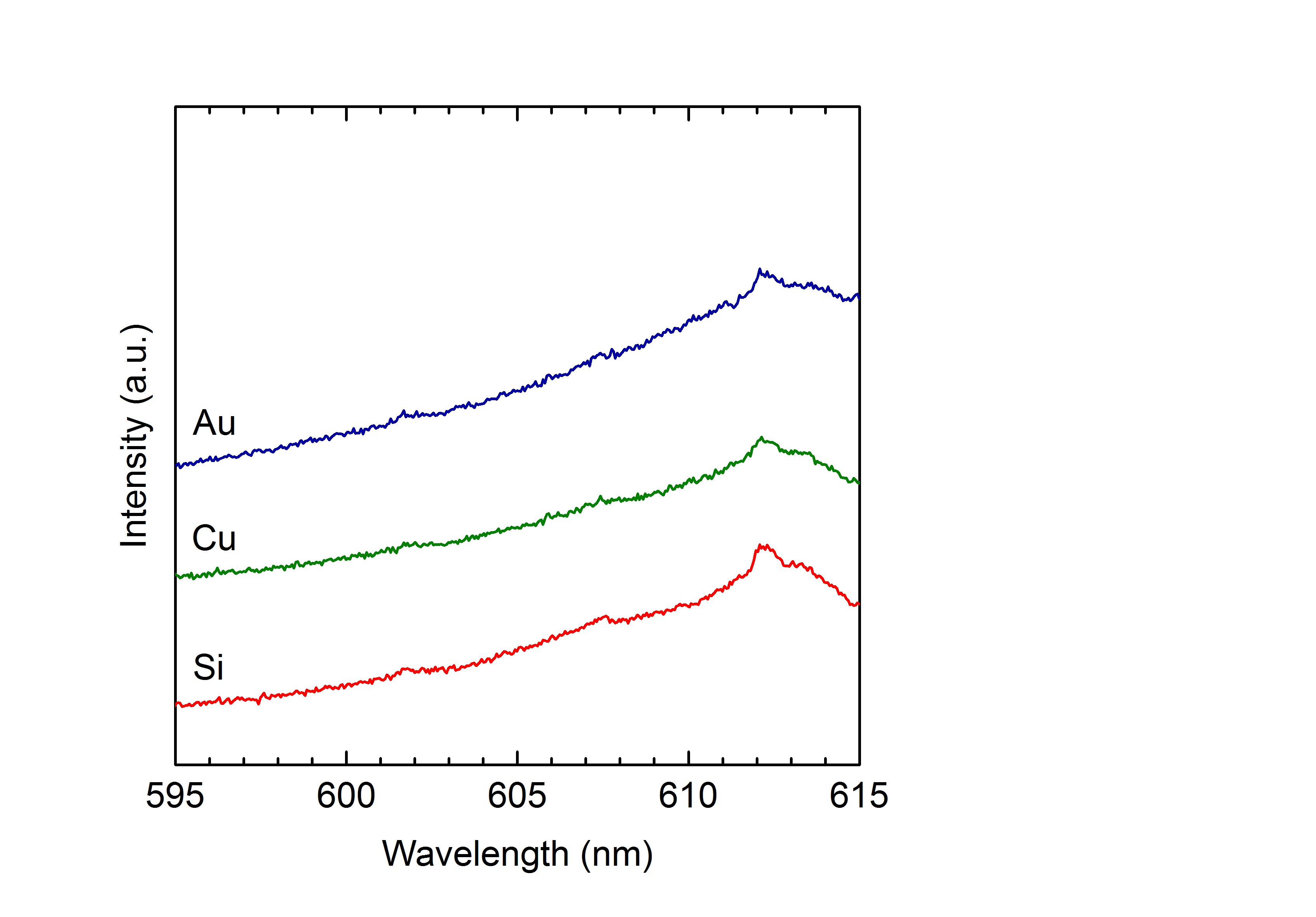


**Figure S2.** PL spectra from diamonds implanted with various elements. The FIB dose and annealing temperature after ion implantation were 2×10^13^ cm^-2^ and 1000 ºC, respectively.

1. **Effect of high-temperature annealing on the formation of the GeV centers.**

The high-temperature anneal has to be performed to fabricate the GeV centers by ion implantation. Ge ion implantation on its own does not produce the characteristic GeV ZPL, while the spectrum after annealing at 800 ºC shows a sharp ZPL. Note that the peaks between 601 and 620 nm observed in the non-treated substrate and implanted sample without annealing are second-order Raman signals from diamond^1^.


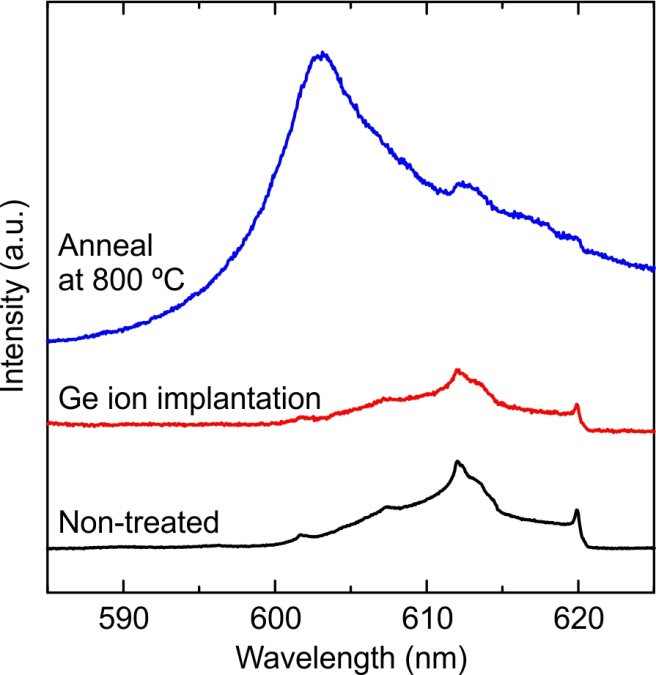


**Figure S3.** Dependence of PL spectra on the preparation process: non-treated single-crystal diamond substrate, after the Ge implantation, and after annealing at 800 ºC.

1. **SIMS profile of CVD-grown diamond film containing GeV centers.**

Figure S4 shows secondary ion mass spectrometry (SIMS) profiles from the diamond film containing the GeV centers incorporated by MPCVD. Since a Ib-type substrate contains a large amount of N atoms, we use it to distinguish the CVD-grown film from the substrate. From the surface (depth=0 nm) to a depth of around 100 nm, the N concentration is under the detection limit. This region corresponds to the CVD film synthesized with a growth rate of 50 nm/h. At the interface at 100 nm, the concentrations of Ge and Si atoms become high due to easier incorporation of impurities at the interface, which is frequently observed for various dopants in diamond^2,3^. Although the Ge concentration fluctuates with depth, the film clearly contains the Ge atoms with a concentration of 1 – 6×10^16^ cm^-3^. In contrast, a higher concentration of Si atoms (~10^18^ cm^-3^) is incorporated in the film from contaminations and/or quartz parts in the growth chamber.


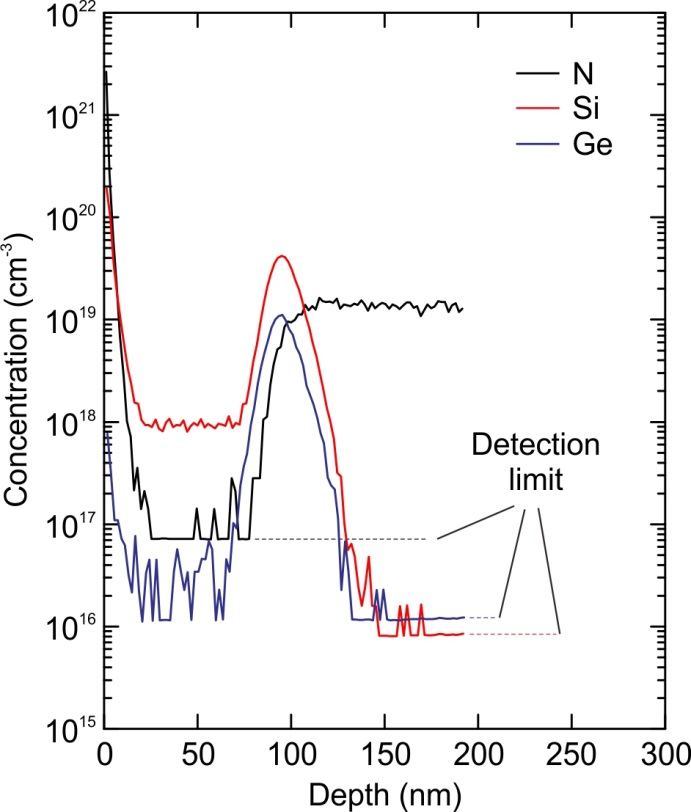


**Figure S4.** SIMS profiles from a diamond film containing GeV centers.

1. **ZPL peak position and intensity of GeV single emitters.**

Eleven single GeV emitters fabricated by ion implantation were measured in this study. We found the considerable variations of the ZPL peak position and ZPL peak intensity in the PL spectra (Fig. S5), which is probably caused by the damages created during ion implantation.


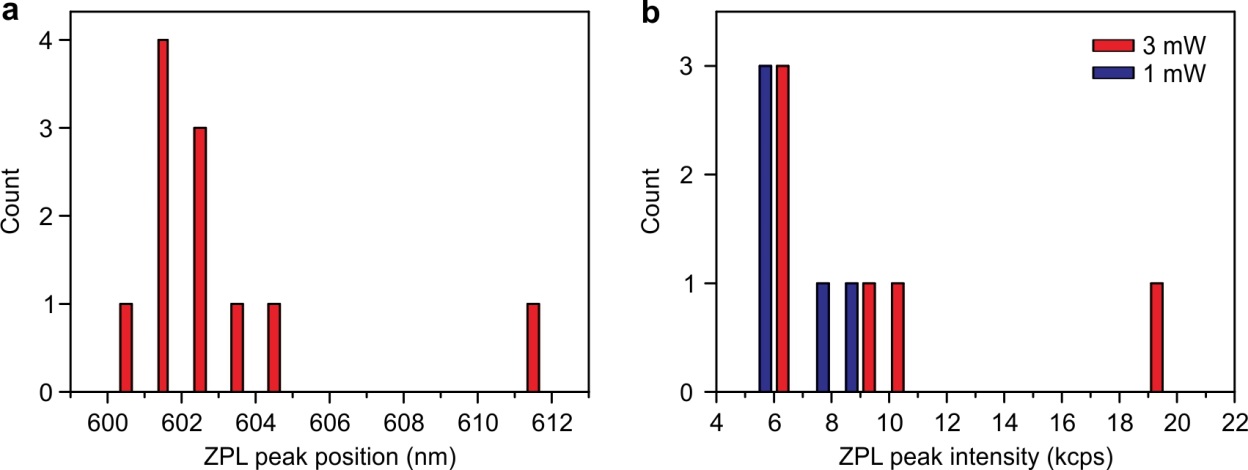


**Figure S5.** Variation of (a) ZPL peak position and (b) ZPL peak intensity of the GeV single emitters. The data were obtained from PL spectra. The peak intensities at the different laser powers are shown separately.

**References**

1. Solin, S. A., Ramdas, A. K. *Phys. Rev. B* **1**, 1687 (1970).
2. Makino, T., Kato., Ri, S-G., Yamasaki, S. & Okushi, H. Homoepitaxial diamond *p-n^+^* junction with low specific on-resistance and ideal built-in potential. *Diamond Relat. Mater.* **17,** 782 (2008).
3. Makino, T., Kato, H., Tokuda, N., Ogura, M., Takeuchi, D., Oyama, K., Tanimoto, S., Okushi, H. & Yamasaki, S. Diamond Schottky-pn diode without trade-off relationship between on-resistance and blocking voltage. *Phys. Status Solidi A* **207,** 2105 (2010).
